# Crohn disease-like enterocolitis remission after empagliflozin treatment in a child with glycogen storage disease type Ib: a case report

**DOI:** 10.1186/s13052-021-01100-w

**Published:** 2021-07-02

**Authors:** Alessandro Rossi, Erasmo Miele, Simona Fecarotta, Maria Veiga-da-Cunha, Massimo Martinelli, Carmine Mollica, Maria D’Armiento, Enza Mozzillo, Pietro Strisciuglio, Terry G. J. Derks, Annamaria Staiano, Giancarlo Parenti

**Affiliations:** 1 Department of Translational Medical Sciences, Section of Pediatrics, University of Naples “Federico II”, Naples, Italy; 2 Section of Metabolic Diseases, Beatrix Children’s Hospital, University Medical Center Groningen, University of Groningen, P.O. Box 30.001, 9700 RB Groningen, The Netherlands; 3 Groupe de Recherches Metaboliques, de Duve Institute, UC Louvain (Université Catholique de Louvain), B-1200 Brussels, Belgium; 4 Section of Medical Imaging, Department of Advanced Biomedical Sciences, University of Naples Federico II, Naples, Italy; 5 Section of Pathology, Department of Advanced Biomedical Sciences, University of Naples Federico II, Naples, Italy; 6 Telethon Institute of Genetics and Medicine, Pozzuoli, Italy

**Keywords:** Glycogen storage disease type Ib, Inflammatory bowel disease, Neutropenia, 1,5-anhydroglucitol, Empagliflozin, Continuous glucose monitoring

## Abstract

**Background:**

Besides major clinical/biochemical features, neutropenia and inflammatory bowel disease (IBD) constitute common complications of Glycogen storage disease type Ib (GSD Ib). However, their management is still challenging. Although previous reports have shown benefit of empagliflozin administration on neutropenia, no follow-up data on bowel (macro/microscopic) morphology are available. We herein present for the first time longitudinal assessment of bowel morphology in a GSD Ib child suffering from Crohn disease-like enterocolitis treated with empagliflozin.

**Case presentation:**

A 14-year-old boy with GSD Ib and severe IBD was (off-label) treated with empagliflozin (20 mg/day) after informed oral and written consent was obtained from the patient’s parents. No adverse events were noted. Clinical symptoms and stool frequency improved within the first week of treatment. Pediatric Crohn disease activity index (PCDAI) normalised within the first month of treatment. Abdomen magnetic resonance imaging (MRI) performed 3 months after treatment initiation showed dramatic decrease in disease activity and length. Similar findings were reported on histology at 5.5 months. At 7.5 months hemoglobin levels normalised and fecal calprotectin almost normalised. Improved neutrophil count, metabolic control and quality of life were also noted. G-CSF dose was decreased by 33% and the patient was partly weaned from tube feeding.

**Conclusions:**

This is the first report presenting extensive gastrointestinal morphology follow-up in a GSD Ib patient receiving empagliflozin. The present case suggests that empagliflozin can be safe and effective in inducing IBD remission in GSD Ib patients and can even postpone surgery. Future studies are required to confirm its effect over time and assess its benefit in various disease stages. The development of an international collaborating networks for systematic data collection is worthy.

**Supplementary Information:**

The online version contains supplementary material available at 10.1186/s13052-021-01100-w.

## Background

Glycogen storage disease type Ib (GSD Ib, MIM#232220) is an inherited disorder of carbohydrate metabolism due to microsomal glucose-6-phosphate transporter (G6PT) deficiency (SLC37A4 gene). The ubiquitously expressed G6PT transports glucose 6-phosphate (G6P) from cytosol to endoplasmic reticulum (ER) where it is oxidized to glucose to ensure glucose homeostasis. G6PT defect results into both glycogenolysis and gluconeogenesis defect [1]. Major clinical features of GSD Ib include fasting hypoglycaemia, hyperlactatemia, hyperuricemia, hyperlipidaemia, hepatomegaly, growth retardation, renal disease [2]. Additionally, GSD Ib patients show neutropenia/neutrophil dysfunction [3] and increased risk of inflammatory bowel disease (IBD) (i.e., Crohn disease-like enterocolitis) [4], and autoimmune disorders [5, 6].

Despite the progress in the (medical and dietary) treatment of GSD Ib over the past years, such immunological complications still heavily impact on patients’ prognosis and quality of life. While evidence regarding the pathogenesis of neutropenia/neutrophil dysfunction and autoimmune disorders has accumulated [7–9], the pathomechanism of IBD in GSD Ib is still unclear; the disturbed immune response may play a role in its pathogenesis [4]. Granulocyte-colony stimulating factor (G-CSF) for neutropenia and conventional drugs for IBD and autoimmune disorders still constitute the current treatment options for most GSD Ib patients. For IBD, conventional treatments are sometimes ineffective and/or associated with side effects and patients might eventually need surgery [1]. Notably, improved prevention/treatment of IBD in GSD Ib ranked as a top priority for research in the international priority setting partnership for liver glycogen storage diseases [10].

Recent evidence has shown a major role for plasma 1,5-anhydroglucitol (1,5AG) in causing neutropenia/neutrophil dysfunction in GSD Ib. 1,5AG enters neutrophils where it is phosphorylated to 1,5-anhydroglucitol-6-phosphate (1,5AG6P). 1,5AG6P is transported by G6PT into the ER, where it is physiologically dephosphorylated. G6PT defect results into cytosolic toxic 1,5AG6P accumulation thus affecting neutrophils survival and function [11].

Empagliflozin is a sodium glucose co-transporter 2 (SGLT2) inhibitor approved for the treatment of type 2 diabetes which reduces renal 1,5AG resorption by increasing urinary glucose excretion. Notably, empagliflozin administration decreased 1,5AG (plasma) and 1,5AG6P (neutrophils) concentrations in GSD Ib mice [11]. Two recent reports have shown same effect in GSD Ib patients with improved neutrophil count/function. Possible benefit on gastrointestinal symptoms have also been reported [12, 13]. However, no follow-up data on bowel (macro/microscopic) morphology are available.

We herein present for the first time longitudinal assessment of bowel morphology in a GSD Ib child suffering from Crohn disease-like enterocolitis treated with empagliflozin.

## Case presentation

### Methods

#### Study design

Empagliflozin is a SGLT2-inhibitor registered and marketed for type 2 diabetes in adults. Its most common adverse effects include low blood pressure and urogenital infections [14]. In the case herein described informed oral and written consent for the off-label treatment with empagliflozin was obtained from the patient and patient’s parents after discussing potential benefits and adverse effects of such treatment. Baseline data were collected 1 (day − 1) or 2 (day − 2) days before starting the treatment during in-hospital admission under medical supervision (day 0). Vital parameters were checked every 2 h within the first 12 h after treatment initiation and subsequently every 8 h. The patient was discharged on day + 5. Regular assessments of his GSD Ib and related conditions were performed at the outpatient clinic every 1 week within the first 2 months of treatment; subsequent evaluations were performed based on the patient’s conditions and medical advice. Blood samples were collected at the maximum distance after last C-GSF administration (day − 2 to day 30: 48 h; day 37 to day 51: 72 h; day 64 to day 71: 96 h; day 78 to day 115: 72 h). Physical examination included: weight, height and body mass index, signs/symptoms of infections, abdominal pain, mouth ulcers and perianal lesions. Adverse events were also recorded. For all results, the specific day of collection (i.e., day before/after starting the treatment) is reported in the main text, tables or figures.

#### Gastrointestinal assessment

An expert endoscopist performed the colonoscopy. During colonoscopy, 4 biopsies were taken from each colonic segment and from the terminal ileum, if entered. The histologic features were assessed by an experienced IBD pathologist, who was blinded to the endoscopic features and clinical history of the patients. Magnetic Resonance Imaging (MRI) was performed by an experienced IBD radiologist (who was blinded to the morphological features and clinical history of the patient) using a high-field (3.0-Tesla) scanner (Trio, Siemens) using a body coil with four channels; the following sequences were acquired: T2-weighted HASTE triggered on the axial plane (TR/TE 2000/91 ms; thickness 6 mm; flip angle 150; matrix: 256 × 157; acquisition time: 64 s), T2-weighted HASTE triggered on the coronal plane (TR/TE 2000/92 ms; thickness 4 mm; flip angle 121; matrix 320 × 256; acquisition time 80 s) with and without fat saturation, T1-weighted in-phase on the axial plane (TR/TE 1500/2.3 ms; thickness 6 mm; flip angle 20; matrix 256 × 154; acquisition time 50 s), T1- weighted out-of-phase on the axial plane (TR/TE 1500/1.37 ms; thickness 3.5 mm; flip angle 20; matrix 256 × 160; acquisition time 58 s) before and after intravenous injection of paramagnetic contrast (gadopentetate dimeglumine, Magnevist, Bayer HealthCare Pharmaceuticals). Disease activity was assessed using the pediatric Crohn disease activity index (PCDAI) [15]. Stool consistency was assessed with the Bristol stool chart.

#### Biochemical tests

Fecal calprotectin was assessed through ELISA assay. Plasma 1,5AG and granulocytes 1,5AG6P were assessed as previously described [12]. Blood (glucose, lactate, cholesterol, triglycerides (TG), uric acid, AST, ALT, albumin, complete blood count, absolute neutrophil count (ANC), C-reactive protein (CRP), erythrocyte sedimentation rate (ESR) within the first hour, creatinine, blood urea nitrogen) and urine tests (creatinine, (24 h-proteins, 24 h-glucose, urinalysis) were performed by using assays with commercially available kits.

#### Glucose monitoring

Besides capillary glucose measurements, flash glucose monitoring (FGM) was performed through an intermittent scanning FGM device (Freestyle Libre2) during the following time frames: 1) baseline to day + 10; 2) + 3 months; 3) + 5.5 months; 4) + 7.5 months; 5) + 8 months. Low-glucose threshold was set at 3.3 mmol/L. In case of glucose concentrations below threshold, capillary glucose was also checked. Hypoglycemia was defined as capillary glucose < 3.3 mmol/L. Due to possible interference of daily life activities, physical activity and the risk of temporary sensor disconnection, both 24-h and night-time (1 a.m. to 5 a.m.) FGM data were analyzed for each time frame by using descriptive statistics. Only days with > 15 time points available were considered for the analysis. Time below range (TBR), time in range (TIR) and time above range (TAR) were defined according to current consensus glucose monitoring recommendations [16].

#### Quality of life (QoL)

Health-related QoL was assessed at baseline and on day + 232 through the Italian version of the Short Form Health Survey (SF-36) questionnaire which has been previously used for GSDI patients. The SF-36 questionnaire consists of 36 items combined into eight scaled scores. The raw score is transformed into a 0–100 scale to generate a summary measure, with higher scores indicating better QoL [17].

### Case presentation

A 14-year-old boy was diagnosed with GSD Ib at age 7 months due to fasting hypoketotic hypoglycemia with high lactate, enlarged liver and neutropenia. The molecular diagnosis of the SLC37A4 gene showed homozygosity for the mutation c.742C > T (p.Gln248X). A diet based on frequent meals and nocturnal gastric drip-feeding was started and the patient was included in a follow-up program at Section of Pediatrics, University of Naples “Federico II”. Several unsuccessful attempts with uncooked cornstarch were made in order to extend his fasting time (all associated with diarrhea, abdominal pain, vomiting). After such attempts, the patient developed avoidant/restrictive food intake disorder. Starting from age 6 years, 24-h gastric drip feeding (GDF) was required. Due to neutropenia, he was also started with i.m. G-CSF.

During the follow-up several complications appeared. At 9 years of age juvenile idiopathic arthritis was diagnosed following the development of arthralgia and arthritis in the right knee and hip. From that age he also experienced recurrent aphthous stomatitis. Since Naproxen (15 mg/Kg/day) administration showed no benefit, i.m. methotrexate (15 mg/m^2^ every 1–3 weeks) was started. At age 13 limitation of range of motion and arthritis in the left knee required intra-articular injection of triamcinolone acetonide. At age 14 hyperuricemia was detected, requiring allopurinol administration (200 mg twice per day). Kidney function was regularly assessed and found normal. Plasma cholesterol (range 1.4–2.5 mmol/L) and TG (range 0.4–1.3 mmol/L) concentrations were constantly decreased.

**Fig. 1:**
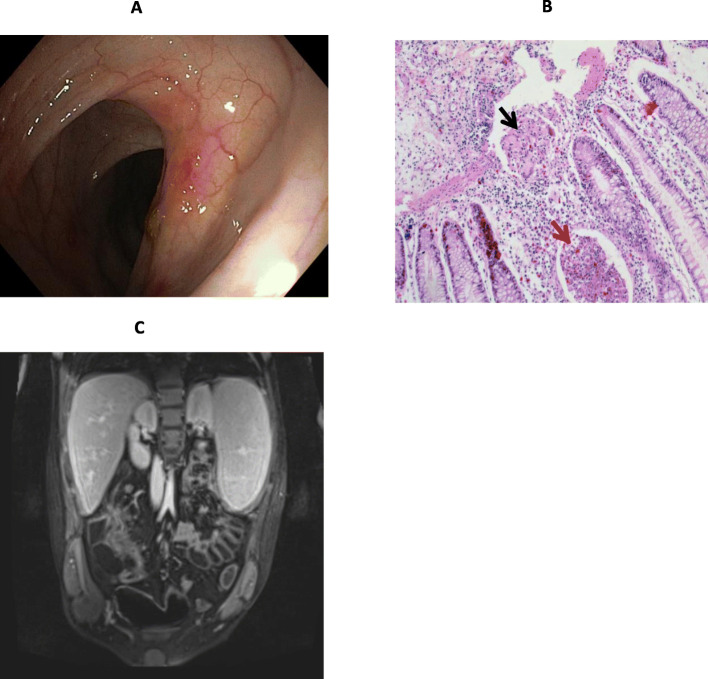
Bowel morphology at baseline. **A** Ileocolonoscopy: ulcerated and ileocecal valve stricture with impossibility to pass through with the scope (Paris classification A1b, L1, B2, G0; SES-CD: 3). **B** Histology (colonic mucosa): architectural irregularity and a mild patchy increase of lamina propria cells with neutrophilic and eosinophilic infiltration, crypt abscesses (red arrow) and an epithelioid cell granuloma (black arrow) indicating active disease. **C** Abdomen MRI: active disease with increased wall thickness (max: 10 mm), diffusion restriction and contrast enhancement in the distal ileum (total length:15–20 cm) and ileal stricture; mesenteric hypertrophy (creeping fat) and lymphadenopathy and conglomerated bowel loops (right lower quadrant) are also shown. *SES-CD: simplified endoscopic score for Crohn’s disease*

Since 10 years of age, he suffered from Crohn-like IBD (PCDAI:75 at the diagnosis). Chronic anemia was also detected requiring several (partly beneficial) intravenous iron infusions (Hb 7.6–10.5 g/dL). Despite methotrexate (15 mg/m^2^ every 1–3 weeks) administration, he experienced 2 disease relapses during the following 3 years. At age 13 switching to adalimumab (40 mg every 2 weeks) was decided after additional bowel and joint relapse. At age 14, further relapse occurred: moderate/severe abdominal pain, 2–5 liquid stools (with mucus) per day, perineal pain due to anal fissure, pain in the left foot with limitation of range of motion due to left metatarsal joints arthritis. No oral lesions were noted. Therefore, the patient was admitted, and extensive reassessment was performed (Table 1). Stricture at the ileocecal valve was detected at ileocolonoscopy (Fig. 1A). Histology showed active disease with crypt abscesses (Fig. 1B). Abdomen Magnetic resonance imaging (MRI) showed active disease with increased wall thickness and contrast enhancement in the distal ileum (total length:15–20 mm) with ileal stricture (Fig. 1C). 7-day ciprofloxacin (2 mg/Kg/day) and metronidazole (15 mg/Kg/day) treatment showed no benefit. Ibuprofen patch was partly effective on arthralgia. Since anti-adalimumab antibodies together with undetectable plasma adalimumab were also detected (Table 1), this treatment was withdrawn (hospital day 5) and ileocecal resection was proposed. 4.8 μg G-CSF/Kg every other day (i.e., 2.4 μg G-CSF/Kg/day) was continued.

**Table 1:** Baseline clinical and biochemical data

	Result	ReferenceRange
**Weight (Kg)**	50	–
**Weight (Z-score)**	−0.46	−2 − + 2
**Height (cm)**	159	–
**Height (Z-score)**	−1.10	−2 − + 2
**BMI**	20	–
**BMI (Z-score)**	0.10	−2 − + 2
**PCDAI**	50	< 10
**Stool consistency type**	6	3–4
**Glucose (mmol/L)**	4.5	3.3–6.1
**Lactate (mmol/L)**	1.8	< 2.2
**Total cholesterol (mmol/L)**	1.4	3.4–5.3
**Triglycerides (mmol/L)**	0.4	0.5–1.6
**Uric acid (mmol/L)**	0.26	0.13–0.39
**AST (U/L)**	12	0–34
**ALT (U/L)**	7	0–55
**Albumin (g/L)**	37	34–48
**Creatinine (mg/dL)**	0.66	0.60–1.10
**Blood Urea Nitrogen (mg/dL)**	13	18–45
**eGFR (ml/min/1.73 m2)**	132.6	100.9–133.3
**White blood cells (WBC)/μL**	3010	5000–15,000
**Neutrophils/μL**	1490	1300–8500
**Lymphocytes/μL**	1370	1300–8500
**Hemoglobin (g/dL)**	8.8	11.5–14.0
**Hematocrit (%)**	33	33–35
**Platelets/μL**	274,000	140,000–440,000
**Fibrinogen (mg/dL)**	233	160–350
**1,5AG (μM)**	155	–
**1,5AG6P (μM)**	1.35	–
**CRP (mg/dL)**	2.8	< 0.5
**ESR (mm/h)**	35	< 20
**Adalimumab (μg/ml)**	< 0.5	5–10
**Anti-adalimumab IgG (ng/ml)**	62.3	< 2.5
**24-h urine protein (mg/24 h)**	< 200	< 200
**24-h urine glucose (mg/24 h)**	not detected	not detected
**Fecal calprotectin (μg/g)**	253	< 100

PCDAI: Pediatric Crohn’s Disease Activity Index; eGFR: estimated glomerular filtration rate; 1,5AG:1,5-anhydroglucitol; 1,5AG6P: 1,5-anhydroglucitol-6-phosphate; CRP: C-reactive protein; ESR: erythrocyte sedimentation rate within the first hour

Off-label treatment with empagliflozin was also discussed with the patient’s family. After oral and written informed consent for this individual treatment, ileocecal resection was postponed, and the patient was started with empagliflozin (day 0, hospital day 16). The starting dose was 5 mg/day (0.1 mg/Kg/day); the dose was further increased to 5 mg twice a day (0.2 mg/Kg/day) on day + 3 and 10 mg twice a day (0.4 mg/Kg/day) on day + 7. Ciprofloxacin was stopped on day − 1; metronidazole was withdrawn on day + 3. The dietary regimen was continued as usual (24-h GDF). No significant changes in vital signs and no serious adverse events were observed. On day + 22 urinary nitrites (with no leukocytes) were detected, with no associated symptoms. Urine culture was ordered and oral cefixime (8 mg/Kg/day) was started (being urinary tract infections common side effects of empagliflozin and considering the risk of metabolic decompensation in case of infection). On day + 27 urine culture tested negative and oral cefixime was withdrawn. Subsequent urinalysis tested normal. 24-h urine glucose was absent on day − 2 and tested constantly increased after treatment initiation (range 13,212–30,775 mg/24 h).

**Fig. 2:**
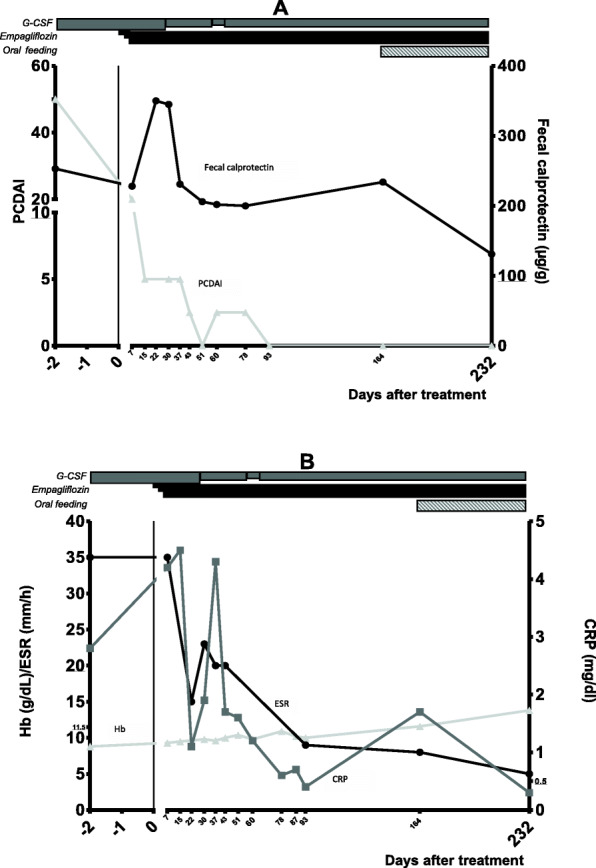
PCDAI and biochemical assessment before and after empagliflozin. **A** PCDAI (light grey triangles) and fecal calprotectin (dark grey circles) values before and after empagliflozin (upper references values for PCDAI (10) and fecal calprotectin (100) are underlined; **B** Hemoglobin (light grey triangles), ESR (Black circle) and CRP (dark grey squares) values before and after empagliflozin (upper reference values for ESR (20) and CRP (0.5) and lower reference value for Hb (11.5) are underlined. *PCDAI: Paediatric Crohn’s Disease Activity Index; CRP: C-reactive protein; ESR: erythrocyte sedimentation rate within the first hour*

Perineal pain and anal fissure improved after 3 days of treatment and disappeared on day + 6. On day + 3 pain in the left foot improved and on day + 5 metatarsal swelling was reduced; starting from day + 20 no signs or symptoms of arthritis were noted. On day + 232 his weight (Z-score: − 0.49), height (Z-score: − 1.03) and BMI (Z-score:0.01) were comparable to baseline. The stool frequency went down to 1–2 x day after 1 week of treatment and 1 x every 2 days after 1 month of treatment. The stool consistency switched from type 6 to type 5 after 2 weeks of treatment and type 4 after 1 month of treatment. The PCDAI decreased from 50 (day − 1) to 20 (day + 7) to 5 (day + 15). Fecal calprotectin increased up to + 40% during the first month of treatment with subsequent decrease. 7.5 months after starting with empagliflozin its value was − 48% compared to baseline and almost normalised (Fig. 2A). Similarly, CRP values increased during the first 2 week of treatment and eventually normalised (occasional spikes occurred). ESR values decreased by 34% after 2 weeks of treatment and normalised after 3 months of treatment. Hemoglobin concentrations constantly increased from the first week of treatment and eventually normalised 5.5 months after starting with empagliflozin (Fig. 2B).

**Fig. 3:**
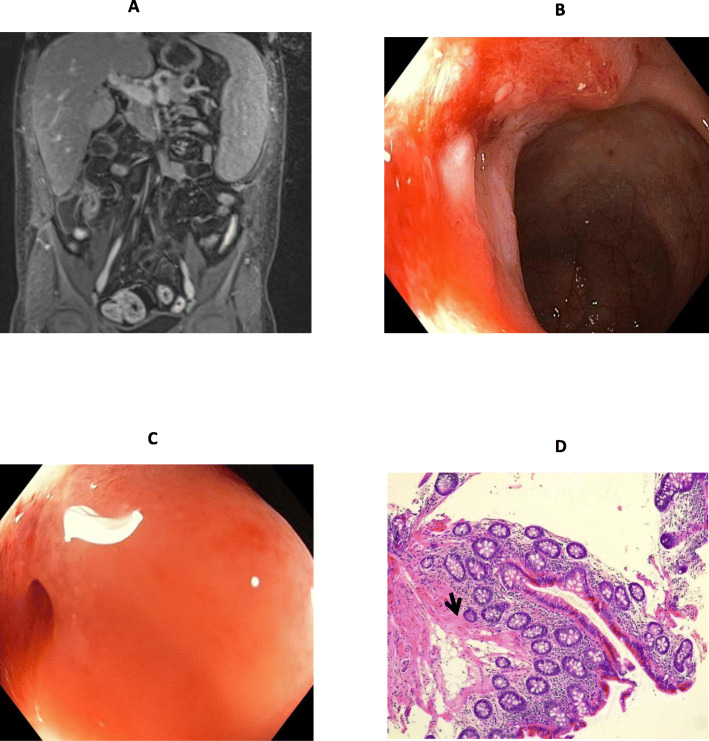
Bowel morphology after empagliflozin treatment. **A** MRI (day + 85): decreased wall thickness (max 6.5 mm), decreased diffusion restriction, decreased contrast enhancement in the distal ileum (total length: 5.5 cm) together with ileal stricture; stable mesenteric hypertrophy (creeping fat) and lymphadenopathy with no evidence of conglomerated bowel loops (right lower quadrant) are also shown. **B**-**C**. Ileocolonoscopy (day + 161): ileocecal valve ulcer and stricture with the impossibility to pass through with the scope (Paris classification A1b, L1, B2, G0; SES-CD: 3). **D** Histology (day + 161, colonic mucosa): minimal architectural distortion, increase of lamina propria, associated with muscularis mucosae hypertrophy (black arrow) and adequate gland representation indicating chronic mild colitis with histologic remission. *SES-CD: simplified endoscopic score for Crohn’s disease*

Abdomen MRI performed at + 85 days showed − 45% wall thickness and − 63% disease length in the distal ileum (total length 5.5 cm) together with ileal stricture (Fig. 3A). At + 161 days ileocolonoscopy showed unchanged stricture at the ileocecal valve (Fig. 3B); histology showed no signs of active disease (Fig. 3C). At the time no significant change in spleen longitudinal diameter was noted (Z-score: baseline: + 8.75; + 161 days: + 7.52).

On day + 163 oral refeeding was proposed. Following discussion with the family, the patient was switched from 24-h (5 mg carbohydrates/Kg/min) to 19-h (4 p.m.-11 a.m.) (5 mg carbohydrates/Kg/min) GDF. In the “GDF-free” hours one morning snack (11 a.m.) and lunch (01.30 p.m.) were included in the hospital setting, providing an overall carbohydrate intake of 2.2 g/Kg (i.e., 7.6 mg carbohydrates/Kg/min). With such a scheme, the patient’s fasting time changed from 0 to 2.5 h during the “GDF-free” hours. Capillary glucose concentrations (checked every 30 min on 3 consecutive days during the 5 h without gastric drip feeding) were > 3.9 mmol/L (range 4.9–7.2 mmol/L). Neither gastrointestinal symptoms/signs nor changes in stool frequency/features were reported during the subsequent 2-month follow-up; on day + 232 fecal calprotectin almost normalised (Fig. 2A).

ANC showed wide variations before empagliflozin (340–4720/μL) with reduced fluctuations (580–2990/μL) after empagliflozin was started. 1,5-AG and 1,5-AG6P concentrations are presented in Additional file 1. The G-CSF dose was gradually decreased and finally set to 4.8 μg G-CSF/Kg every 3 days (i.e. 1.6 μg G-CSF/Kg/day). Lactate concentrations stayed within the reference range (1.3–2.3 mmol/L). A slight increase in cholesterol and TG concentrations (which reached the reference range) was observed (Additional file 2A). Uric acid concentrations stayed normal and allopurinol was gradually discontinued (Additional file 2B). Liver and kidney function were regularly checked and tested normal.

Capillary glucose values were below < 3.3 mmol/L (range 2.6–3.3 mmol/L) on 20/32 low-glucose events measured by FGM within the first week of treatment and promptly increased upon glucose administration via the feeding tube. No signs or symptoms of hypoglycemia were reported. Occasional (2–4 times per month) asymptomatic mild hypoglycemias (range 2.7–3.3 mmol/L) occurred during the subsequent 6-month capillary glucose self-monitoring. Data on FGM monitoring are shown in Additional file 3. A substantial decrease in TBR as well as an increase in TIR were observed (also after oral refeeding was started). The patient’s QoL score improved from 37.64 (baseline) to 74.44 (day + 232).

## Discussion and conclusions

The management of IBD is still challenging in GSD Ib as its pathogenesis remains [1, 3]. Conventional treatments (i.e., corticosteroids, immunomodulators, biological agents) are sometimes ineffective and/or associated with side effects (e.g. leucopenia, anemia, diarrhea) and patients might eventually need surgery [18]. Being the cornerstone of treatment for neutropenia, G-CSF has also led to IBD remission in some GSD Ib patients [19]. However, its efficacy is variable and long-term G-CSF administration may cause side effects (e.g. enlarged spleen) and increased risk for malignancies (e.g. acute myeloid leukemia, myelodysplastic syndrome) [20, 21]. Therefore, more effective treatments are required to improve patients prognosis and quality of life.

Although previous reports have shown possible benefit of empagliflozin on gastrointestinal symptoms [12, 13], follow-up data on bowel (macro/microscopic) morphology are not available. In the case herein reported, we presented comprehensive gastrointestinal assessment in a child with GSD Ib, showing clear benefit of empagliflozin administration on Chron disease-like enterocolitis. No symptomatic hypoglycemias and no adverse events were associated with empagliflozin administration.

Clinical improvement was noted within the first week of treatment and eventually led to normal stool frequency/consistency and PCDAI normalisation. Clinical remission occurred within the first month of treatment. Biochemical improvement occurred within the first 2 weeks of treatment and remission was documented after 2 months of treatment. Strikingly, the benefit on clinical picture and hemoglobin concentrations appeared as soon as the end of the first week. Morphology studies also showed partial IBD remission within 3–6 months of treatment. Notably, a dramatic improvement in the disease length and activity was documented on the abdomen MRI after 3 months of treatment as well as histologic remission after 5.5 months of treatment. Empagliflozin allowed to postpone ileocecal resection (and possibly decrease the length of the bowel segment to be resected) in the present case. However, no major endoscopic changes were noted 5.5 months after starting treatment. Those data show that empagliflozin may be effective in healing the inflammatory lesions/strictures but might not be able to reverse fibrotic strictures once established. Such conclusion suggests early empagliflozin administration in GSD Ib patients with IBD before the onset of (irreversible) intestinal fibrosis. 0.4 mg/Kg/day empagliflozin were administered in the present case. Previous study showed 0.3–0.7 mg/Kg/day to lay within the therapeutic window for neutropenia [12]. It is still unclear if higher doses can be more effective or whether a specific dose range might exert special benefit on other disease complications (e.g., IBD, arthritis). Future studies should address this issue.

Besides the effect on IBD, decreased 1,5AG and 1,5AG6P concentrations as well as higher/more stable neutrophil count were also documented after starting with empagliflozin. Based on those data, the G-CSF dose was decreased by 33% in the present case. Undoubtedly, reducing G-CSF dose can decrease the risk of side effects and malignancies associated with its long-term administration. However, not all GSD Ib patients treated with empagliflozin are able to discontinue G-CSF [12]. The reason for such discrepancy is still unclear. Possible role of additional factors contributing to empagliflozin response (e.g., genotype, renal function, glycosylation status) should be further addressed for optimal patient selection. The results herein reported also support possible role of disrupted immune response in the pathogenesis of IBD in GSD Ib. Indeed, a role for 1,5AG and 1,5AG6P in modulating other peripheral blood mononuclear cells has been postulated [11].

Despite 33% reduction in G-CSF dose, no change in spleen size was observed in the current patient. In 3 out of the 5 previous GSD Ib patients that have been previously described to be treated with empagliflozin and who presented with splenomegaly, 2 showed decreased spleen size only 9 months after starting empagliflozin (G-CSF was discontinued or decreased by 81%, respectively). Despite G-CSF discontinuation, 1 patient showed stable spleen size 3 months after starting empagliflozin [12]. Longer follow-up studies are warranted to clarify when to expect benefit on splenomegaly.

Additionally, improved metabolic control was noted in the present case. Increase of (low) cholesterol and TG levels and reversal of hyperuricemia (leading to allopurinol discontinuation) were detected after empagliflozin administration. Likely, normalisation of plasma cholesterol and TG were secondary to intestinal healing in the present case. FGM data showed stable glucose levels and eventually no hypoglycemia was detected. Strikingly, the patient also experienced (limited) amount of time in the “above-range” (Additional file 3). Such findings are in line with previous report [12] suggesting that IBD might concur to metabolic control in GSD Ib patients by limiting intestinal glucose absorption. Interestingly, recent research has shown the impact of life-long diet on gut microbiota in GSD Ib [22]. FGM may constitute an additional, minimally invasive monitoring tool for GSD Ib patients.

Not only led empagliflozin to improved clinical conditions and biochemical/morphological markers but also allowed drug dose reduction/discontinuation in the present case. As a matter of fact, the patient was switched from 4-drug (i.e., adalimumab, allopurinol, G-CSF, ibuprofen patch) to 2-drug (G-CSF, empagliflozin) regimen with a simplified drug schedule. Notably, the number of painful G-CSF injections was decreased. Benefits on healthcare costs (empagliflozin is less expensive than G-CSF and biologic therapies for IBD) as well as reduced healthcare use by GSD Ib patients can also be expected. Indeed, the gross monthly financial burden for medication decreased by 59% in the present case (3586 € vs 1467€).

Improved QoL was also observed after empagliflozin administration. Patients with IBD show increased prevalence of psychological disturbances like depression and anxiety [23]. In the present case, the patient agreed on restarting oral feeding after 8 years, allowing (in part) weaning from tube feeding. This result suggests that, by improving the IBD-related symptoms, empagliflozin can also exert a positive effect on psychosocial health and well-being in patients with GSD Ib.

Although renal function was constantly normal in the present case, future studies should assess the effect of empagliflozin on renal function in GSD Ib patients [24, 25].

Overall, the present case shows that empagliflozin administration is safe and effective in inducing IBD remission in GSD Ib patients and can postpone surgery. It also improves neutrophil count and metabolic control. Since this is the first case documenting comprehensive longitudinal IBD morphology follow-up in a patient with GSD Ib treated with empagliflozin, future studies are needed to confirm its safety and efficacy over time and assess its benefit in various disease stages. As empagliflozin has the potential to change the natural history and management of GSD Ib patients, the development of an international collaborating networks for systematic data collection on its safety and efficacy is worthy.

## Supplementary Information

**Additional file 1. **Neutrophil count (light grey triangles), 1,5AG (black circles) and 1,5AG6P (dark grey squares) before and after empagliflozin. Plasma 1,5-AG concentration dropped from ±250 μM before treatment to ±50 μM after 2 weeks on empagliflozin. Concentration of 1,5-AG stayed relatively constant until day + 164, before a change in the diet introducing a daily oral intake of carbohydrates. On day + 232, approximately 2 months after this change, plasma 1,5-AG was only very slightly increased to 60 μM. After treatment, 1,5-AG6P present in leukocytes and measured in whole blood samples was reduced by 4- to 5-fold when compared to values before starting empagliflozin. *1,5AG: 1,5-anhydroglucitol; 1,5AG6P: 1,5-anhydroglucitol-6-phosphate*.

**Additional file 2. **(A) Plasma cholesterol (grey triangles) and TG (black circles) before and after empagliflozin (reference values for cholesterol (3–5) and TG (0.5–1.5) are underlined); (B) Plasma uric acid concentrations before and after empagliflozin (reference values are underlined). *TG: triglycerides*.

**Additional file 3.** Flash glucose monitoring data.

## Data Availability

The datasets used and/or analysed during the current study are available from the corresponding author on reasonable request.

## Abbreviations

ANC: Absolute neutrophil count
CRP: C-reactive protein
ESR: Erythrocyte sedimentation rate
FGM: Flash glucose monitoring
GSD Ib: Glycogen storage disease type Ib
IBD: Inflammatory bowel disease
PCDAI: Pediatric Crohn disease activity index
QoL: Quality of life
TG: Triglycerides
1,5AG: 1,5-anhydroglucitol
1,5AG6P: 1,5-anhydroglucitol-6-phosphate
