# Supplementary figures and images for: Crohn disease-like enterocolitis remission after empagliflozin treatment in a child with glycogen storage disease type Ib: a case report
Source: Ital J Pediatr. 2021 Jul 2;47:149. doi: 10.1186/s13052-021-01100-w (PMC8254289; doi:10.1186/s13052-021-01100-w)

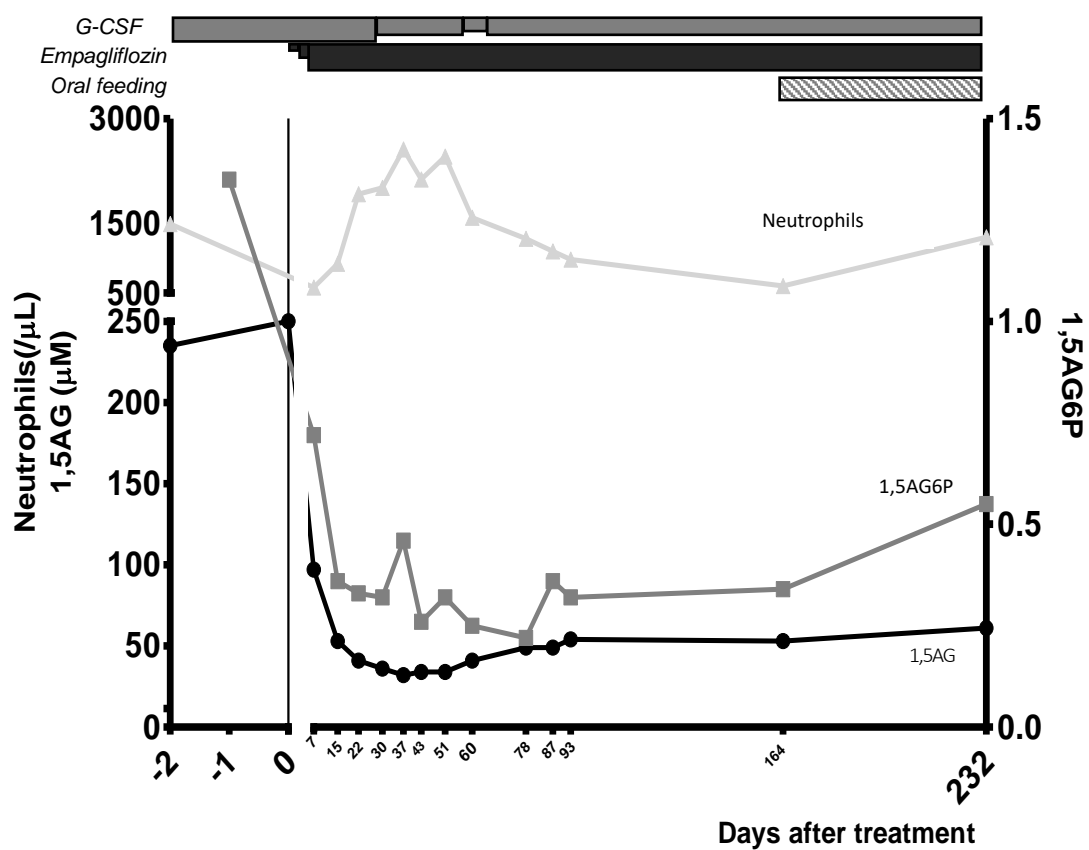

Additional file 1

Supplement: Supplementary file 1 — Additional file 1. Neutrophil count (light grey triangles), 1,5AG (black circles) and 1,5AG6P (dark grey squares) before and after empagliflozin. Plasma 1,5-AG concentration dropped from ±250 μM before treatment to ±50 μM after 2 weeks on empagliflozin. Concentration of 1,5-AG stayed relatively constant until day + 164, before a change in the diet introducing a daily oral intake of carbohydrates. On day + 232, approximately 2 months after this change, plasma 1,5-AG was only very slightly increased to 60 μM. After treatment, 1,5-AG6P present in leukocytes and measured in whole blood samples was reduced by 4- to 5-fold when compared to values before starting empagliflozin. 1,5AG: 1,5-anhydroglucitol; 1,5AG6P: 1,5-anhydroglucitol-6-phosphate. [file 13052_2021_1100_MOESM1_ESM.pdf]

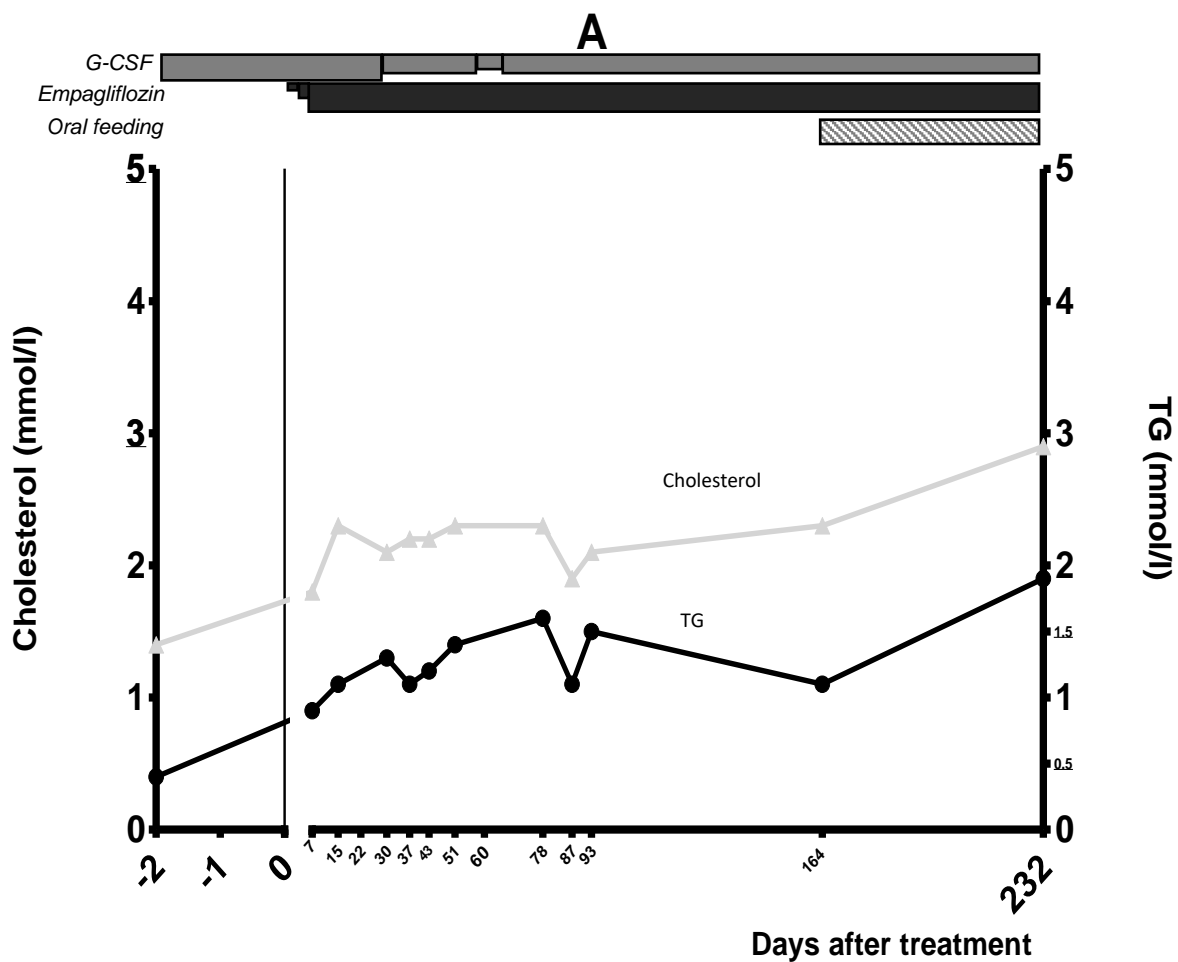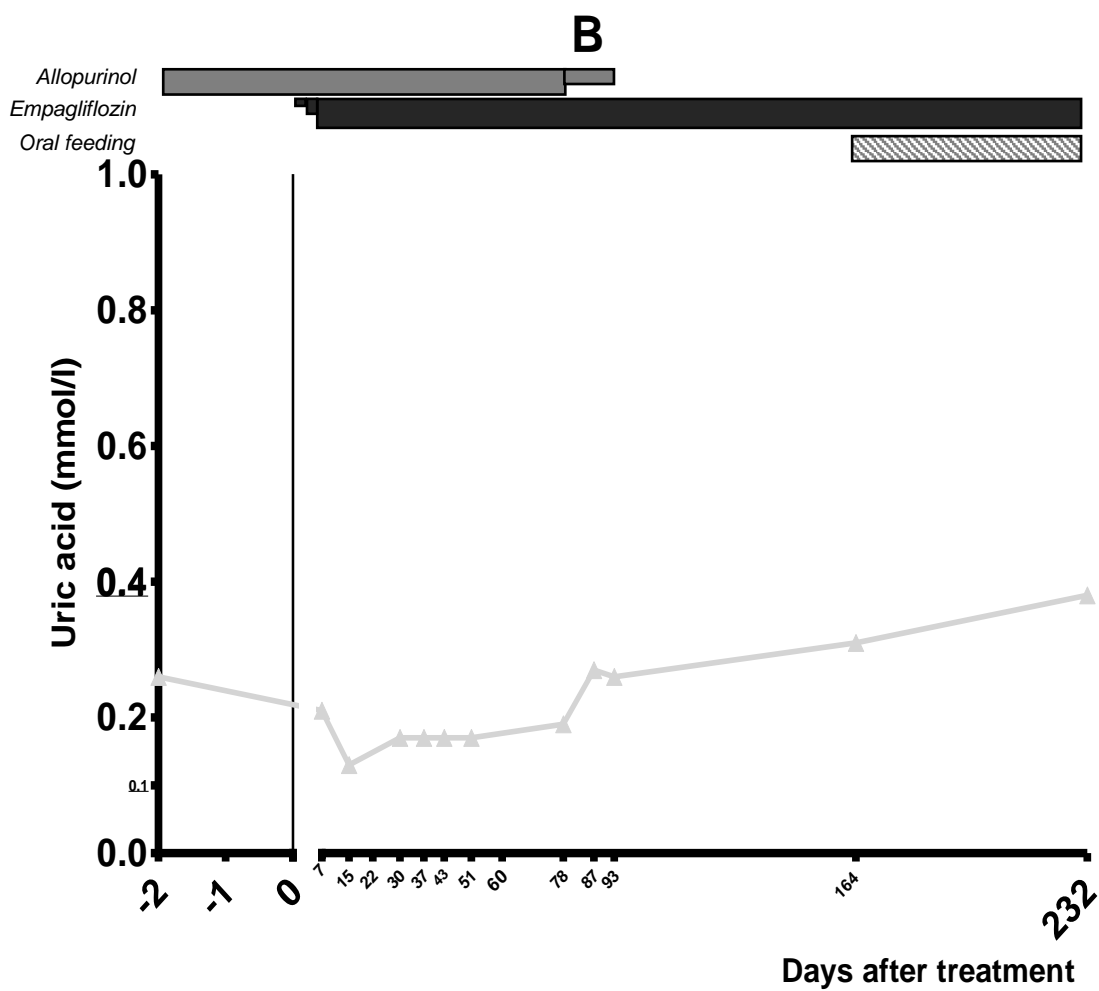

**Additional file 2**

Supplement: Supplementary file 2 — Additional file 2. (A) Plasma cholesterol (grey triangles) and TG (black circles) before and after empagliflozin (reference values for cholesterol (3–5) and TG (0.5–1.5) are underlined); (B) Plasma uric acid concentrations before and after empagliflozin (reference values are underlined). TG: triglycerides. [file 13052_2021_1100_MOESM2_ESM.pdf]
