# Supplementary material for: Crohn disease-like enterocolitis remission after empagliflozin treatment in a child with glycogen storage disease type Ib: a case report
Source: Ital J Pediatr. 2021 Jul 2;47:149. doi: 10.1186/s13052-021-01100-w (PMC8254289; doi:10.1186/s13052-021-01100-w)
Supplement: Supplementary file 3 — Additional file 3. Flash glucose monitoring data. [file 13052_2021_1100_MOESM3_ESM.pdf]

										TIME BELOW RANGE (TBR)		TIME IN RANGE (TIR)		TIME ABOVE RANGE (TAR)	
**Time frame**	**Study day**	**Days analysed**	**Time points analysed**	**median**	**min**	**max**	**variance**	**SD**	**Coefficient of Variation**	**<3.0 mmol/L**	**≥ 3 < 3.9 mmol/L**	**≥ 3.9 ≤ 7.8 mmol/L**	**≥ 3.9 ≤ 10 mmol/L**	**> 7.8 mmol/L**	**>10 mmol/L**
				mmol/L	mmol/L	mmol/L	mmol/L	mmol/L	%	%	%	%	%		
24-h	-1 to +10	12	1151	4.44	2.61	7.33	14.52	0.90	1.11	3,2	25,5	71,2	71,2	0,0	0,0
	+85 to +96	12	794	5.17	2.67	8.67	25.74	1.20	1.30	2,4	16,4	80,5	81,2	0,8	0,0
	+164 to +176	13	1178	5.55	2.94	8.50	18.07	1.00	1.00	0,1	5,8	93,3	94,1	0,8	0,0
	+214 to +226	13	1175	5.44	2.89	9.78	18.54	1.02	1.00	0,6	5,9	92,2	93,5	1,4	0,0
	+245 to +257	13	1174	5.94	2.94	10.33	23.07	1.13	1.10	0,1	3,5	91,7	96,3	4,7	0,2
Night	-1 to +10	12	195	4.78	2.94	6.94	16.87	0.97	1.11	1,0	17,9	81,0	81,0	0,0	0,0
	+85 to +96	12	195	5.61	2.94	7.89	29.78	1.29	1.32	1,5	13,3	83,6	85,1	1,5	0,0
	+165 to '+176	12	193	5.44	3.17	7.44	14.69	0.90	0.93	0,0	6,7	93,3	93,3	0,0	0,0
	+215 to +226	12	192	5.44	3.17	7.28	12.84	0.84	0.87	0,0	6,3	93,8	93,8	0,0	0,0
	+246 to +257	12	194	6.06	3.00	8.33	17.87	1.00	0.92	0,0	3,6	93,8	96,4	2,6	0,0

Additional file 3.
